# Assessment of the Potential Impact of Aqueous and Ethanolic Malt Extracts on HepG2 Liver Cells

**DOI:** 10.17113/ftb.64.03.26.9286

**Published:** 2026-09-15

**Authors:** Diana Santos, Maria João Pereira, Ângelo Jesus, Rita Ferraz de Oliveira, Ana Isabel Oliveira, Cláudia Pinho

**Affiliations:** 1 ESS, Polytechnic of Porto, Rua Dr. António Bernardino de Almeida 400, 4200-072 Porto, Portugal; 2 REQUIMTE/LAQV, ESS, Polytechnic of Porto, Rua Dr. António Bernardino de Almeida 400, 4200-072 Porto, Portugal

**Keywords:** craft beer, malt, antioxidant activity, metabolic activity, alanine aminotransferase (ALT)

## Abstract

**Research background:**

Malt is the second most abundant raw material in beer production and the main source of phenolic compounds. However, information on the specific contribution of different types of malt to the biological activity of beer and their effects on liver function remains limited. Therefore, this study aims to evaluate the antioxidant potential and effects on liver function of a Portuguese craft beer (imperial stout, IS-N) and the aqueous and ethanolic extracts of the malts present in IS-N beer (Carafa III, Caramunich III, Carapils and Pilsner).

**Experimental approach:**

Aqueous and ethanolic extracts of malt were obtained by solid-liquid extraction at a ratio of 1 g of powder to 9 mL of distilled water and 1 g of powder to 10 mL of *φ*(ethanol)=95 %, respectively. Total phenolic content (TPC) was determined by the Folin-Ciocalteu method, and antioxidant activity was evaluated by ABTS, DPPH and metal chelating capacity (ferrozine) assays. Cytotoxicity was evaluated in HepG2 cells using the MTT assay after exposing the cells to different concentrations of the samples (1–500 µg/mL) for 24 and 48 hours. Liver function was evaluated by determining alanine aminotransferase (ALT) activity in the cell supernatant.

**Results and conclusions:**

TPC values, expressed as gallic acid equivalents (GAE), ranged from (7.1±0.5) to (28.2±0.5) mg/g, with the aqueous extract of Caramunich III ((23.6±0.6) mg/g) standing out for its high TPC and greater antioxidant capacity (IC_50_ ABTS=(17.3±0.8) µg/mL and IC_50_ DPPH=(152.6±9.3) µg/mL). Malt extracts showed no cytotoxicity up to 250 µg/mL, while IS-N beer with 8.5 % ethanol showed cytotoxicity at 500 µg/mL after 24 hours. IS-N beer induced lower ALT amounts than single malts, suggesting a possible synergistic effect between its bioactive compounds. Thus, evaluating the biological activity of malts could contribute to the production of beers with a better functional profile and reduced hepatic impact.

**Novelty and scientific contribution:**

This study breaks new ground by exploring the role of malts in the antioxidant activity and hepatic effects of craft beer, providing new data that could guide the development of beverages with a functional profile that is more beneficial to health.

## INTRODUCTION

Beer is the most popular alcoholic beverage in the world, widely consumed and with an estimated global production, in 2023, of around 1.88 billion hectolitres per year (*1*). Historically, beer has been valued not only for its sensory characteristics, but also for its cultural and social role in different societies.

In recent decades, the craft beer sector has experienced remarkable growth (*1*), driven by consumers who value products with unique sensory profiles, differentiated composition, sustainable appeal and potential health benefits (*1*, *2*). The basic composition of beer includes four main ingredients: water, malt, hops (*Humulus lupulus* L.) and yeast (*3*). However, the final product can be enriched with other ingredients that confer specific sensory and functional properties (*4*).

Among the essential components of the traditional formulation, malt and hops play central roles in both the sensory quality and bioactive profile of beer. Hops impart characteristic bitterness and aroma, contribute to foam stability and have antimicrobial action that aids in the preservation of the beverage (*5*). In addition to these technological functions, hops contain resins, essential oils, and a variety of phenolic compounds with recognised antioxidant activity, representing approximately one third of the phenolic compounds present in beer (*5*, *6*).

Malt, usually obtained from barley (*Hordeum vulgare* L.), also contributes decisively to the flavour, aroma, colour and body of the beverage (*7*). In addition, malt is an important source of phenolic compounds with recognised antioxidant activity, contributing about two thirds of the phenolic content of beer. The composition of these compounds varies according to the type of cereal used, the degree of roasting and the malting process (*8*, *9*). Thus, the chemical diversity between different types of malt can affect not only the sensory properties of beer, but also its physiological effects, including its impact on liver function (*10*).

The liver is a critical organ responsible for multiple functions, including detoxification, and metabolism of exogenous substances, such as ethanol and bioactive compounds present in beer (*10*). As the main site of alcohol metabolism, it is particularly exposed to toxic by-products generated during this process (*11*). Among these substances are acetaldehyde and reactive oxygen species (ROS), which can cause cellular damage and compromise liver function when produced in excess. Although moderate beer consumption has been associated with beneficial effects such as antioxidant capacity, neuroprotective properties, improved lipid profile, reduced risk of atherosclerosis and decreased inflammatory markers (*12*, *13*), excessive beer consumption is associated with hepatotoxicity, which can lead to hepatic steatosis, alcoholic hepatitis, fibrosis and cirrhosis (*10*). Hepatotoxicity is often assessed through the analysis of serum biomarkers, especially the enzymes alanine aminotransferase (ALT) and aspartate aminotransferase (AST), whose elevation indicates damage to the integrity of liver cells (*14*).

Thus, this work aims to characterise the extract of a Portuguese craft beer and its malts (aqueous and alcoholic extracts) in terms of their antioxidant potential and impact on liver function, by analysing cell toxicity and the activity of the ALT enzyme in human hepatocarcinoma cells (HepG2 cells).

## MATERIALS AND METHODS

### Chemicals

Gallic acid and Folin-Ciocalteu reagent were purchased from Merck (Darmstadt, Germany). Absolute ethanol, disodium phosphate, and ethylenediaminetetraacetic acid (EDTA) were supplied by VWR Chemicals, Avantor (Solon, OH, USA). Quercetin was obtained from Sigma-Aldrich, Merck (St. Louis, MO, USA), while dipotassium peroxydisulfate came from Biochem (Cosne-Cours-sur-Loire, France). Ferrozine, 2,2'-azino-bis(3-ethylbenzothiazoline-6-sulfonic acid) (ABTS) and 1 % antibiotic/antifungal solution were purchased from Thermo Scientific, Thermo Fisher Scientific (Kandel, Germany). Monosodium phosphate was supplied by J. T. Baker (Deventer, the Netherlands), and dimethyl sulfoxide (DMSO) and sodium chloride were obtained from Fisher Scientific (Loughborough, UK). The 3-(4,5-dimethyl-2-thiazolyl)-2,5-diphenyl-2H-tetrazolium bromide (MTT) came from Acros Organics (Geel, Belgium), while sodium carbonate was purchased from Atom Scientific (Manchester, UK). The 6-hydroxy-2,5,7,8-tetramethylchroman-2-carboxylic acid (Trolox) was obtained from TCI (Zwijndrecht, Belgium). Trypsin, phosphate-buffered saline (PBS) and minimum essential medium (MEM) were supplied by Corning (Manassas, VA, USA), and foetal bovine serum (FBS) was obtained from Biochrom KG (Berlin, Germany). Finally, the ALT enzyme assay kit was purchased from Randox Laboratories (Crumlin, Ireland).

### Beer and malt samples

Portuguese craft beer imperial stout (IS-N) (Porto, Portugal) was chosen because of its potential antioxidant and hepatoprotective activity demonstrated in our previous study (*15*), and also because of the availability of its malts. IS-N beer is made with Carafa Special III, Caramunich III, Carapils and Pilsner malts (selected malts) provided by the brewery that produces the craft beer (IS-N).

### Preparation of beer and malt extracts for analysis

The malt samples were prepared as described by Mareček *et al.* (*16*) and Wu *et al.* (*17*), with slight modifications. The malt cereals were ground in an electric mill (Taurus^®^; Aromatic, Oliana, Spain) for 40 s and then sieved through a 500 μm mesh sieve. To obtain the aqueous malt extract, 25 g of powder was added to 225 mL of distilled water and placed in a water bath at 45 °C for 15 min. After cooling, the mixture was filtered through Whatman filter paper No. 1 and the filtrates obtained were stored at −80 °C until completely frozen. The extracts were then freeze-dried (LABCONCO^®^; FreeZone^®^, Kansas City, MO, USA) at 0.007 kPa, with a condenser surface temperature of −72 °C for 3 days and stored at −80 °C.

To obtain the ethanolic malt extract, 1 g of powder was added to 100 mL of 95 % (*V/V*) ethanol solution. The extraction was carried out at room temperature by magnetic stirring (985VW0CHSEUA; VWR^®^, Avantor, Leuven, Belgium) at 400 rpm for 30 min. The mixture was then filtered by gravity (Whatman filter paper No. 1) and the filtrates obtained were placed in a rotary evaporator (RV8; IKA^®^, Staufen, Germany) under reduced pressure (90 kPa), at 60 rpm and a controlled temperature of 40 °C (HB10; IKA^®^), until complete ethanol evaporation. The extracts were freeze-dried under the same conditions described above.

The IS-N craft beer with *φ*(ethanol)=8.50 %, was provided by the brewery responsible for its production. Upon receipt, the sample underwent the necessary treatments prior to experimental testing, following the procedures described in our previous study (*15*). The contents of the bottle were initially mixed uniformly for 10 s and then subjected to gas removal by sonication (Bandelin Sonorex^®^, Bandelin, Berlin, Germany) for 40 min at a frequency of 35 kHz at room temperature. The alcohol was then removed using a rotary evaporator (RV8; IKA^®^) operating at 40 °C, 60 rpm and a pressure of 90 kPa for 1 h. The sample was then freeze-dried (LABCONCO^®^; FreeZone^®^) under a pressure of 0.007 kPa, with the condenser surface cooled to −72 °C for three days.

### Determination of total phenolic content and antioxidant capacity

The total phenolic content (TPC) by the Folin-Ciocalteu method and antioxidant activity using the 2,2'-azino-bis(3-ethylbenzothiazoline-6-sulfonic acid) (ABTS) and ferrozine (metal chelating activity) assays in aqueous and ethanolic extracts of malts under study were determined as described in our previous study (*15*).

For TPC determination, 250 μL of the sample (1 mg/mL), distilled water (blank) or standard gallic acid solutions (5–100 μg/mL) were mixed with 2.5 mL of 0.2 M Folin-Ciocalteu reagent and incubated for 5 min at room temperature in the dark. Subsequently, 2 mL of sodium carbonate solution (75 g/L) were added, and the final volume was adjusted to 5 mL with distilled water. After 1 h of incubation under the same conditions, the absorbance was measured at 760 nm in a UV-Vis spectrophotometer (model UV-1600PC; VWR, Avantor, Leuven). The total concentration of phenolic compounds was determined by comparison with the standard curve of gallic acid, and the results were expressed in milligrams of gallic acid equivalents (GAE) per gram of sample.

For ABTS assay, 2.45 mM of dipotassium peroxydisulfate solution was added to 7.0 mM ABTS and the reaction mixture was kept in the dark for 16 h at room temperature to form ABTS radical. The obtained solution was diluted in PBS until it reached an absorbance of 0.70±0.05 at 734 nm. Next, 0.3 mL of the sample (malt extracts from IS-N craft beer), Trolox standard (1–1000 μg/mL) or distilled water (blank) were added to 2.7 mL of the ABTS^+^˙ solution. After 30 min of incubation at room temperature in the dark, the absorbance was measured at 734 nm in a UV-Vis spectrophotometer (model UV-1600PC; VWR, Avantor, Leuven). Antioxidant activity was expressed as average inhibitory concentration IC_50_ (μg/mL) and the percentage of inhibition was calculated according to the established formula:


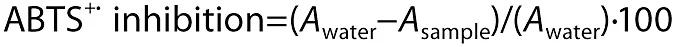
 /1/

In the ferrozine assay, 50 μL of sample (beer or positive control, EDTA) were added to a 0.15 mM Fe(II) sulfate solution and left to stand for 5 min, protected from light. Next, 50 μL of 0.5 mM Fe(II) cyanide were added, the mixture was shaken vigorously and left for 10 min at room temperature protected from light. The absorbance was measured at 562 nm using a microplate reader (Multiskan FC; Thermo Scientific, Thermo Fisher Scientific, Woodlands, Singapore). Metal chelating activity was expressed as IC_50_ (μg/mL), and the percentage inhibition was calculated according to the following formula:


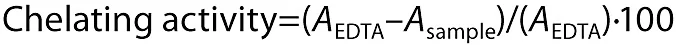
 /2/

The antioxidant potential using the 2,2-diphenyl-1-picrylhydrazyl assay (DPPH) was determined as described by Silva *et al.* (*18*), with slight modifications. Briefly, 19.4 μL of sample (1, 5, 10, 25, 50, 100, 250 and 500 μg/mL of malt extracts, craft beer (IS-N), or quercetin as positive control) was added to 175 μL of light-protected DPPH radical (100 μM). The absorbance was measured in a microplate reader (Multiskan FC; Thermo Scientific, Thermo Fisher Scientific) at a wavelength of 520 nm. The readings were repeated every minute for 1 hour. The ability to neutralise the DPPH radical was expressed as IC_50_ (μg/mL) and the percentage of inhibition was calculated using the following formula:


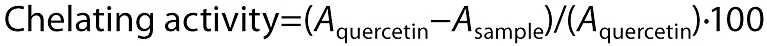
 /3/

### Cell line maintenance

HepG2 cells were maintained as described by Oliveira *et al.* (*19*). The cells were cultured in 25 cm^3^ flasks in MEM medium supplemented with 10 % (*V*/*V*) FBS and 1 % (*V*/*V*) antibiotics (ampicillin and streptomycin) and incubated at 37 °C with 5 % CO_2_ (AL01-01-100; Advantage-Lab^®^, Schilde, Belgium). The culture medium was changed every 2 days, and subcultured when 60/80 % confluence was reached, with the addition of 0.25 % trypsin-EDTA.

### Evaluation of liver toxicity

To study the cytotoxicity of craft beer, aqueous malt extracts and malt extracts prepared with 95 % (*V*/*V*) ethanol, the methods described by Carvalho *et al.* (*20*) and Viegas *et al.* (*21*) were used, with slight modifications. Briefly, HepG2 cells were incubated in 96-well plates (VWR^®^, Avantor, Radnor, PA, USA) at a density of 2.0·10^5^ cell/well, 48 h before incubation with the samples. The cells were then treated with different concentrations of the samples (1−500 μg/mL) for 24 and 48 h (100 µL final volume/well), and cytotoxicity was estimated using the MTT assay. After the incubation period, 10 µL of MTT solution (5 g/mL) were added to each well and left for 1 h in an atmosphere of 5 % CO_2_ at 37 °C (AL01-01-100; Advantage-Lab^®^). The medium was then removed and the formed formazan crystals were dissolved in a *φ*(DMSO,ethanol)=50 % solution. Absorbance was measured at 570 nm using a microplate reader (Multiskan FC; Thermo Scientific, Thermo Fisher Scientific, Woodlands). The results were expressed as a percentage of cell viability relative to the control (cells without extract) using the following equation:


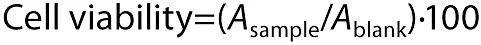
 /4/

### Alanine aminotransferase activity measurement

Alanine aminotransferase (ALT) enzyme activity was measured using the method described by González *et al.* (*22*), with assay kits obtained from Randox Laboratories. HepG2 cells were treated with aqueous and ethanolic extracts of the malts, and with craft beer (IS-N) in aqueous, and ethanolic (*φ*=8.5 %) solvents. Concentrations were selected based on cell viability results, choosing, for each sample, the concentration that induced the highest and lowest cell viability. After incubation for 24 and 48 h, the supernatant was removed from the wells, and enzyme activities were determined immediately. Results were expressed in international units per litre (IU/L).

### Statistical analysis

Statistical analysis was carried out using GraphPad Prism^®^ v. 8.0 software (*23*). The results of the assays were analysed in triplicate and expressed as mean±standard deviation. The TPC and antioxidant potential of the samples were compared using a one-way analysis of variance (ANOVA) with Tukey's multiple comparison test for pairwise comparisons. The correlation between the mean TPC and the antioxidant assays was determined by the correlation coefficient (r), calculated by Pearson's product-moment correlation.

For the cell viability assay, the one-way ANOVA with Dunnett's multiple comparison test was used to compare each value with the control (cells without extract). The metabolic activity of the aqueous and ethanolic extracts of each malt and beer sample at the same concentration was compared using Sidak's multiple comparison test (one-way ANOVA). The same test was used to compare the levels of liver enzymes produced by incubating the cells with two concentrations of the same extract. For all assays, statistical differences were considered significant when p*<*0.05.

## RESULTS AND DISCUSSION

### Total phenolic content

Malt is the main source of phenolic compounds in beer (75–80 %) (*18*). Phenolic compounds are secondary metabolites containing at least one aromatic ring linked to hydroxyl groups or other structural elements. These compounds represent a group of substances with different chemical structures, differing in their resistance to free radicals and metal chelation, as well as other reactions that occur in beer or living cells (*5*). Recognised as potent antioxidants, phenolic compounds play critical roles in the sensory properties, colour and colloidal stability of beer, and also contribute to its antioxidant activity (*24*, *25*). Table 1 shows the results of the TPC determined in the four malts. The TPC values of craft beer (IS-N) were determined in our previous study (TPC as *w*(GAE)=(8.3±0.2) mg/g) (*15*).

**Table 1:** Results of total phenolic content (TPC) in malt extracts

Malt extract	TPC as *w*(GAE)/(mg/g)
Carafa III aqueous	(22.2±1.1)^abc^
Carafa III ethanolic	(20.7±0.4)^ab^
Caramunich III aqueous	(23.6±0.6)^ac^
Caramunich III ethanolic	(28.2±0.5)^d^
Carapils aqueous	(14.0±0.9)^e^
Carapils ethanolic	(7.1±0.5)^f^
Pilsner aqueous	(10.9±0.3)^g^
Pilsner ethanolic	(8.8±0.2)^h^

Different letters in superscript indicate statistically significant differences (p*<*0.05). GAE=gallic acid equivalents

In this study, the TPC, expressed as GAE, of malts varied considerably between the extracts analysed ((7.1±0.5)–(28.2±0.5) mg/g). The ethanolic extract of Caramunich III had the highest value ((28.2±0.5) mg/g) and was statistically superior to the others (p*<*0.05), followed by the aqueous extract of the same malt. On the other hand, the ethanolic extract of Carapils had the lowest TPC ((7.1±0.5) mg/g), significantly lower than the others (p*<*0.05). These results indicate that the amount of TPC changes the malt colour, showing that darker malts such as Caramunich III and Carafa III have a higher mass fraction of phenolic compounds than lighter malts such as Pilsner and Carapils.

At the European level, the colour of malts and beer is expressed in units of the European Brewery Convention (EBC). Pilsner malt (2.5 and 4 EBC, data from Sr Cervejeiro Online Brewery Store) is the lightest of the malts analysed and is classified as a base malt, used mainly to provide fermentable sugars because of its high enzymatic activity (*18*, *26*, *27*). Meanwhile, Carapils (10−20 EBC), Caramunich III (140−160 EBC) and Carafa III (1250−1400 EBC, for all three malts data available from Sr Cervejeiro Online Brewery Store) are considered specialty malts (*27*). Due to the heat treatment they undergo, a loss of enzymatic activity is observed and for this reason these malts are traditionally used in smaller quantities (usually around 5 %) than base malts (*26*). Their main function is to contribute to specific sensory characteristics of the beer, such as colour, aroma and flavour (*18*, *26*).

The variation in TPC observed between different malts and types of extract (aqueous *vs* ethanolic) corroborates the literature, which highlights the influence of the solvent on the extraction of phenolic compounds (*9*, *28*). Other factors, such as the barley variety, the growing region, the use of fertilisers, and the germination and drying stages, also have an impact on the phenolic content and antioxidant activity of malts (*26*, *29*, *30*). The main phenolic compounds present in malt are (+)-catechin, protocatechuic acid, quercetin, ferulic acid and gallic acid (*30*).

According to the literature, the amount of phenolic compounds in malt tends to increase its colour intensity, especially up to around 450-500 EBC (*31*). This is partly due to the polymerisation and preservation of phenolic compounds during moderate drying regimes. In addition, products of the Maillard reaction, such as melanoidins, also contribute to the antioxidant potential and are formed in greater quantities during drying and roasting. However, in malts with a colour above 500 EBC, such as the Carafa III analysed in this study, there is a decrease in the content of phenolic compounds, which can be explained by the ability of melanoidins to retain simple phenolic compounds in their structure, as well as the possible inactivation of enzymes (such as ferulic acid esterase) responsible for their release from barley cell walls (*31*).

The literature also shows variations in TPC between different types of malt and barley. Zhao *et al.* (*9*) reported TPC values for dry barley, expressed as GAE, ranging from 2.17 to 2.56 mg/g among 14 varieties of Chinese malted barley (Gan4, Gan3 and Wupi1, Ken2 and Ken3, Humai8, Humai16, Gangpi1, Suyin1, Huaimai19, Linnong, Nongmai, KA4B and Gang2), extracted with 80 % acetone (*V*/*V*). In the study by Gąsior *et al.* (*26*), the wort obtained from Pilsner malt had a TPC of (192.6±8.7) mg/L. In contrast, in the study by Neto *et al.* (*32*), the aqueous extract of Pilsner malt revealed a considerably lower TPC of only (0.91±0.00) µg/mL.

In the study conducted by Šimić *et al.* (*33*), which evaluated nine varieties of barley and their respective malts (Barun, Bravo, Bingo, Premium, Vanessa, Tiffany, Maxim, Gazda and Rex), extraction with acidified methanol (*V*(HCl):*V*(methanol)=1:100) demonstrated a higher content of total phenolics and higher antioxidant activity in malts than in their corresponding barleys. The TPC values on dry mass basis for the malts ranged from (1.53±0.09) to (1.82±0.00) mg/g, while for the barleys they ranged from (1.27±0.03) to (1.67±0.09) mg/g. These results are in accordance with the study conducted by Dvořáková *et al.* (*29*), who found that most of the ten aqueous extracts from the malts studied (from barley varieties Prestige, Jersey, KM 1910, KM 2084, Malz, Merlin, Sebastian, Tolar, Bojos and Amulet) had on dry mass basis higher antioxidant activity than their respective barleys, ranging from 1.1 to 2.9 mg/g and from 0.6 to 1.5 mg/g, respectively.

In our previous study (*15*), IS-N beer had a TPC of extract (8.3±0.2) mg/g, which was lower than that observed in the malt extracts analysed, with the exception of the ethanolic extract from Carapils. This difference may be related to technological brewing processes, such as malting, the milling method, and the method and mode of mashing, which affect the final phenolic content of the wort and beer (*26*, *31*). Moreover, the difference may also be related to the fact that, in beer formulation, dark (or special) malts are traditionally used in smaller quantities than the base malt (in this case, Pilsner), which has lower TPC values (*26*). This may explain the lower TPC values observed in beer than in the malts analysed individually.

The study carried out by Censi *et al.* (*28*) corroborates the differences in TPC between the different extracts used and the final beer. In the aqueous starter malts, TPC ranged from (11.7±1.8) to (16.568±2.412) mg/g, and in the ethanolic extracts it ranged from (28.1±1.0) to (72.1±1.9) mg/g. The beers analysed ranged from (19.0±1.1) to (35.8±0.15) mg/g.

The phenolic profile of beer is diverse, encompassing catechins and proanthocyanidins, prenylchalcones and their flavanone derivatives, as well as flavonols, hydroxybenzoic acids, hydroxycinnamic acids and stilbenes (*34*).

It should be noted that the Folin-Ciocalteu method, although widely used for drinks and plant extracts, is not specific to phenolic compounds and can be affected by other compounds with reducing activity (*9*, *25*). Therefore, the possibility of overestimating the TPC results cannot be ruled out (*25*).

### Antioxidant assays

Beer and malts represent a heterogeneous matrix containing antioxidants with different mechanisms of action, which results in different contributions to their antioxidant capacity (*35*, *36*). The antioxidant activity of the imperial stout craft beer (IS-N) under study was determined previously by our research group using different colorimetric antioxidant assays (ABTS and ferrozine assays) (*15*), as there is no standard method that can objectively characterise the total antioxidant capacity of the samples (*37*).

ABTS and DPPH assays allow the antioxidant potential of an extract to be measured by its ability to donate hydrogen atoms and electrons, neutralising the respective radicals with a consequent decrease in absorbance (*38*). Ferrozine assay, based on metal-chelating activity, estimates the antioxidant potential of the extract by its ability to chelate Fe(II), preventing their oxidation to Fe(III) ions, and thus inhibiting the formation of the hydroxyl radical (OH˙), the initiator of the oxidative chain (*39*). Some assays, such as ABTS and DPPH, are commonly used in beers and cereals due to their sensitivity, convenience and simplicity (*33*). The results of the assays in malts are shown in Table 2. Regarding the antioxidant activity of the craft beer (IS-N) determined by the DPPH assay, it was not possible to determine IC_50_ values for the range of concentrations analysed.

**Table 2:** Antioxidant activity of malt extracts with ABTS, DPPH and ferrozine assays

Malt extract	ABTS (IC_50_ µg/mL)	DPPH (IC_50_ µg/mL)	Ferrozine (IC_50_ µg/mL)
Carafa III aqueous	(40.3±0.9)^aeh^	ND	ND
Carafa III ethanolic	(118.2±4.9)^b^	(229.3±9.7)^ac^	ND
Caramunich III aqueous	(17.3±0.8)^c^	(152.6±9.3)^b^	ND
Caramunich III ethanolic	(133.59±2.01)^d^	(227±7)^ac^	ND
Carapils aqueous	(41.7±0.1)^aeh^	ND	ND
Carapils ethanolic	(546.9±5.3)^f^	(441±9)^d^	ND
Pilsner aqueous	(57.1±1.2)^g^	ND	ND
Pilsner ethanolic	(38.2±0.8)^aeh^	ND	ND

ABTS=2,2’-azino-bis(3-ethylbenzothiazoline-6-sulfonic acid) assay, DPPH=2,2-diphenyl-1-picrylhydrazyl assay, IC_50_=concentration able to inhibit by 50 %. The different letters indicate statistically significant differences (p*<*0.05). ND=not detected IC_50_ at the tested concentrations

According to the ABTS assay, the antioxidant potential of the malt extracts ranged from (17.3±0.8) µg/mL (Caramunich III aqueous extract) to (133.6±2.0) µg/mL (Caramunich III ethanolic extract). It should be noted that the aqueous extract of Caramunich III had the lowest IC_50_ value, showing high antioxidant capacity (IC_50_<50 µg/mL), with significant differences compared to other samples (p*<*0.05).

Analysis of the results confirms that the aqueous malt extracts generally showed greater antioxidant potential (from (17.3±0.8) µg/mL for Caramunich III to (57.1±1.2) µg/mL for Pilsner) than the ethanolic extract (from (38.2±0.8) µg/mL for Pilsner to (547±9) µg/mL for Carapils). This suggests that the antioxidant compounds with the greatest potential to neutralize 50 % of the initial amount of ABTS^+^ free radicals are more soluble in water than in 95 % ethanol, except for the ethanolic extract of Pilsner malt (activity of the ethanolic extract higher than the aqueous extract, p*<*0.05). The study conducted by Censi *et al.* (*28*) corroborates these results, as the aqueous extracts of the initial malt of the analysed beers showed a higher antioxidant potential, expressed as TE, ranging from (21.4±2.7) to (46.82±0.031) µmol/g, determined by the ABTS assay, than the ethanolic extracts with 70 % ethanol, with an antioxidant potential ranging from (41.3±2.8) to (97±19) µmol/g.

In our previous study (*15*), IS-N craft beer showed an IC_50_ of (80.1±1.1) µg/mL determined by the ABTS assay, indicating moderate antioxidant activity (50<IC_50_<100 µg/mL) (*40*), similar to that found in the present work for the ethanolic extracts of Carafa III and Caramunich III and the aqueous extract of Pilsner.

In the study by Gąsior *et al.* (*26*), the wort obtained from Pilsner malt inhibited (22.2±0.9) % of ABTS^+^ radicals. Also, in the study by Oliveira Neto *et al.* (*32*), (42.8±13.3) μL of aqueous extract from Pilsner malt neutralised 50 % of ABTS^+·^ radicals. Zhao *et al.* (*9*) found that the antioxidant potential, expressed as TE, of *φ*(acetone)=80 % extracts from malts from 14 Chinese varieties of barley, determined by the ABTS assay, ranged from 11.39 to 13.58 µmol/g of dry barley. Finally, Dvořáková *et al.* (*29*) demonstrated that the antioxidant capacity, expressed as GAE, of aqueous malt extracts from 10 varieties, determined on dry extract basis using the ABTS assay, ranged from 0.20 to 0.45 mg/g.

Regarding the DPPH assay, the antioxidant potential of the malt extracts ranged from (152.6±9.3) μg/mL in the aqueous extract of Caramunich III to (441±9) μg/mL in the ethanolic extract of Carapils. The aqueous extract of Caramunich III was the only aqueous extract for which it was possible to determine the IC_50_ value in the range of concentrations tested (1–500 μg/mL). However, its antioxidant capacity is considered low, given that its IC_50_>100 μg/mL (*40*).

On the other hand, as previously mentioned, it was not possible to determine IC_50_ in the IS-N craft beer, in the aqueous extract of Carafa III, or either of the Pilsner malt extracts at the concentrations tested (1–500 μg/mL), indicating low antioxidant efficacy as determined by the DPPH method.

In the study by Özcan *et al.* (*41*), methanolic extracts from malt showed greater antioxidant activity than the extracts obtained with the same solvent from barley, with inhibition percentages of (67.31±0.00) % for malt and (66.48±0.00) % for barley. These results corroborate those obtained by Šimić *et al.* (*33*), who, using acidified methanol extraction, reported DPPH inhibition ranging from (63.1±1.9) to (68.4±2.0) % for malts and from (58.2±2.2) to (65.2±1.5) % for barley.

The data obtained in this study do not corroborate the results observed by Silva *et al.* (*18*), in which the beers showed better antioxidant activity than the raw materials. In the present study, aqueous extracts of Carafa III, Caramunich III, Carapils and Pilsner had greater antioxidant activity than the beer (IS-N), in the ABTS assay. Using the DPPH assay, ethanolic extracts of Carafa III and Carapils, as well as ethanolic and aqueous extracts of Caramunich III, demonstrated greater antioxidant potential than the beer.

These differences in the results can be attributed to various biotic and abiotic factors that influence plant physiology and the production of secondary metabolites with antioxidant activity, as well as the type of solvent used for extraction, which plays a crucial role in the efficiency of phenolic compound extraction (*9*).

In addition, the selection of barley varieties with greater metal-chelating activity is essential for beer quality, as it contributes to the stability of its flavour. This is because transition metal ions can activate the oxygen present in the drink, promoting oxidation reactions that result in the formation of compounds responsible for undesirable flavours (*9*).

In the present study, using the ferrozine assay, it was not possible to determine the IC_50_ values within the range of concentrations tested (1–500 μg/mL) for the craft beer or the malt extracts. These results indicate that the samples studied had low antioxidant capacity (IC_50_>100 μg/mL) (*40*).

In the study conducted by Zhao *et al.* (*9*), metal-chelating activity values, expressed as ethylenediaminetetraacetic acid equivalents (EDTAE), in Chinese malts extracted with 80 % acetone varied between 1.15 and 2.06 μmol/g dry barley, with a weak correlation between TPC and metal-chelating capacity. In the study by Silva *et al.* (*18*), the chelating activity of metals in the aqueous extracts of the evaluated malts ranged from (12.0±0.5) to (24.8±0.6) %, with the aqueous extract of Carapils malt showing a chelating percentage of (20.5±0.7) %.

In this study, for malt extracts, there was a significant correlation only between the ABTS and DPPH antioxidant assays (p*<*0.05) when analysing the ethanolic extracts (Table 3), as would be expected given that these assays are based on the transfer of electrons or hydrogen atoms. Furthermore, there was a negative correlation between TPC and the ABTS assay in the aqueous extracts, as well as between TPC and the ABTS and DPPH assays in the ethanolic extracts, which confirms the fact that phenolic compounds contribute to the elimination of ABTS and DPPH radicals, *i.e.* antioxidant capacity.

**Table 3:** Correlation between total phenolic content (TPC) and antioxidant assays in malt extracts

	TPC aqueous extract	TPC ethanolic extract	ABTS aqueous extract	ABTS ethanolic extract	DPPH aqueous extract	DPPH ethanolic extract
TPC aqueous extract	1.000	-	-0.841	-	-	-
TPC ethanolic extract	-	1.000	-	-0.451	-	-0.940
ABTS aqueous extract	-	-	1.000	-	-	-
ABTS ethanolic extract	-	-	-	1.000	-	0.999*
DPPH aqueous extract	-	-	-	-	-	-
DPPH ethanolic extract	-	-	-	-	-	1.000

TPC=total phenolic content, ABTS=ABTS^+^˙ neutralisation assay, DPPH=2,2-diphenyl-1-picrylhydrazyl assay. *Correlation is significant at the 0.05 level (two-tailed)

In the study conducted by Zhao *et al.* (*9*), a weak correlation was observed between the TPC of malt extracts and metal-chelating capacity, but a significant correlation of TPC with the ABTS and DPPH assays (p*<*0.01), as well as a significant correlation (p*<*0.01) between the ABTS and DPPH assays themselves, as also observed in the present study. In turn, the study by Gąsior *et al.* (*26*) reported a strong correlation between the TPC of malt extracts and the ABTS assay (r=0.75).

### Toxicity of craft beer and malt extracts

#### Metabolic activity

In the present study, HepG2 cells were incubated with IS-N beer in aqueous and ethanolic solvents corresponding to the original alcohol percentage (8.5 % (*V*/*V*)), as well as with the aqueous and ethanolic malt extracts, for 24 and 48 h, in order to assess their cytotoxicity (cell viability of less than 80 %) (Fig. 1 and Fig. 2) and the amount of ALT enzyme (Fig. 3) (*42*, *43*).

**Fig. 1:**
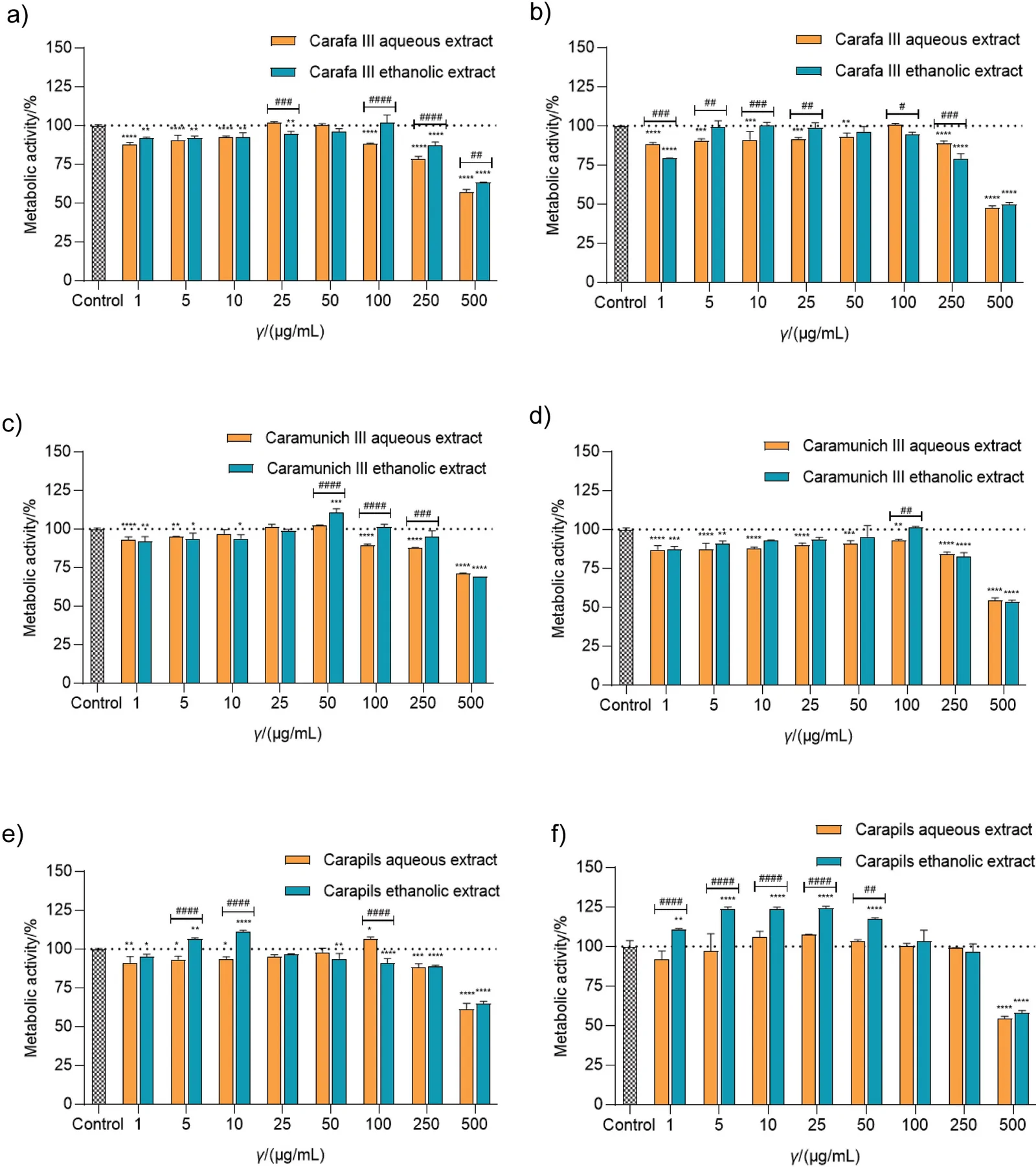
Cytotoxicity, determined by the MTT assay, of different concentrations of aqueous and ethanolic extracts of: a and b) Carafa III after 24 and 48 h, c and d) Caramunich III after 24 and 48 h, and e and f) Carapils malt after 24 and 48 h, respectively, in HepG2 cells. Data are presented as mean±standard deviation of three independent samples (*N*=3), in triplicate. Values *p<0.05, **p<0.01, ***p<0.001 and ****p<0.0001 when compared to the control (cells without extract) and #p<0.05, ##p<0.01, ###p<0.001 and ####p<0.0001 when comparing two similar concentrations

**Fig. 2:**
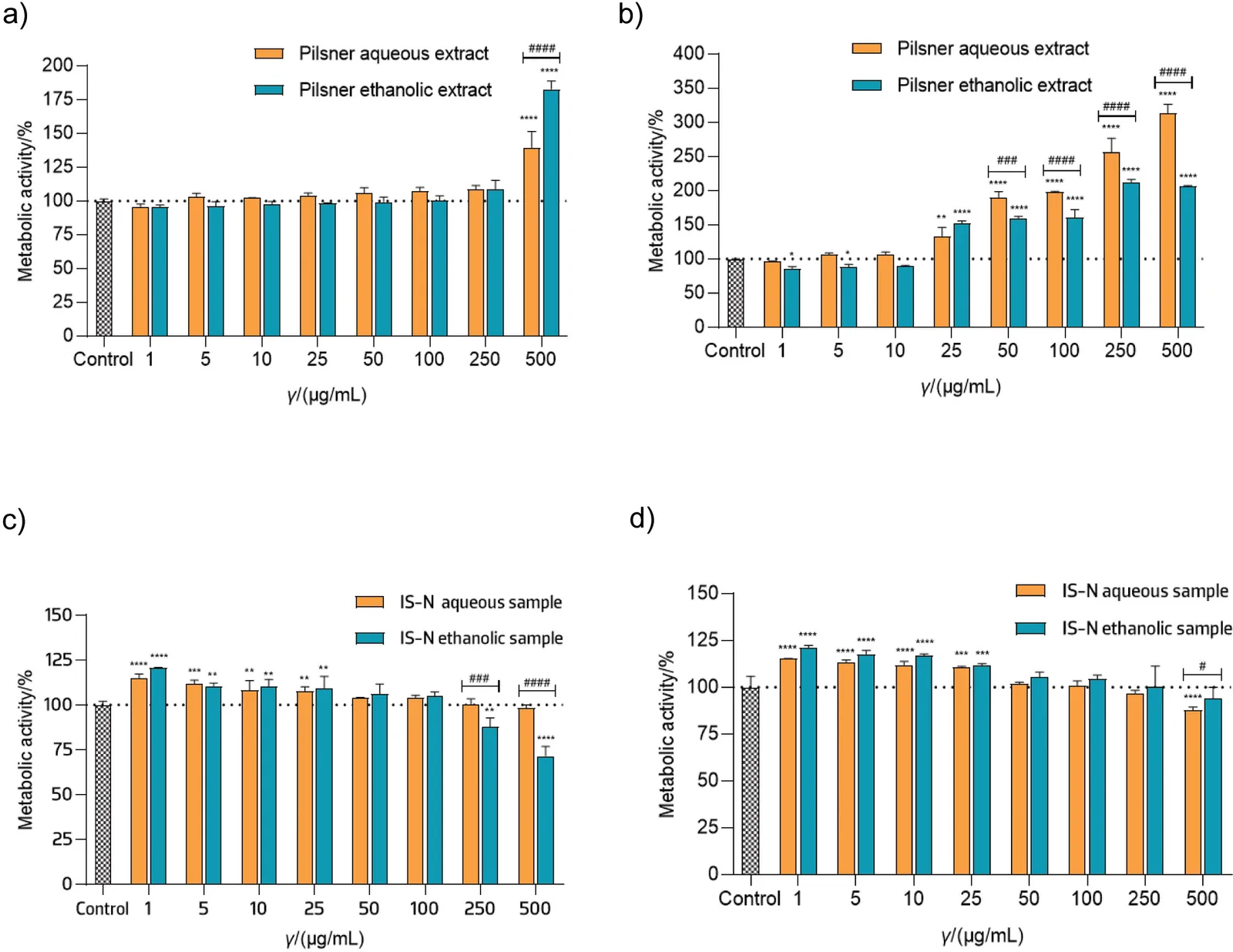
Cytotoxicity, determined by the MTT assay, of different concentrations of aqueous and ethanolic extracts of: a and b) Pilsner malt after 24 and 48 h, and c and d) IS-N craft beer without ethanol (aqueous sample) and with 8.5 % ethanol (ethanolic sample) after 24 and 48 h, respectively, in HepG2 cells. Data are presented as mean±standard deviation of three independent samples (*N*=3), in triplicate. Values *p<0.05, **p<0.01, ***p<0.001 and ****p<0.0001 when compared to the control (cells without extract) and #p<0.05, ##p<0.01, ###p<0.001 and ####p<0.0001 when comparing two similar concentrations

**Fig. 3:**
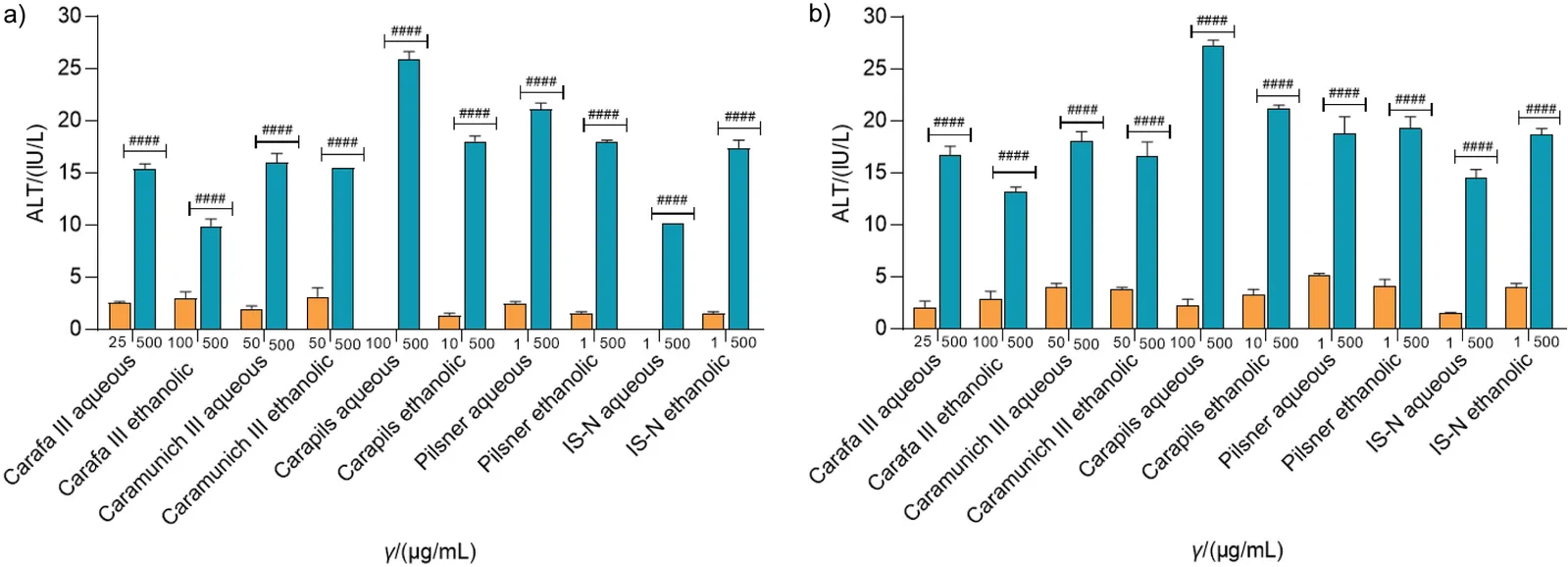
*In vitro* determination of ALT of different concentrations of IS-N beer and aqueous and ethanolic extracts of malts in HepG2 cells. Aqueous and ethanolic extracts of malts and IS-N craft beer without ethanol (aqueous sample) and with 8.5 % ethanol (ethanolic sample) after: a) 24 h and b) 48 h. Data are presented as mean±standard deviation of three independent samples (*N*=3), in triplicate. Values #p<0.05, ##p<0.01, ###p<0.001 and ####p<0.0001 when comparing different concentrations of the same sample

The HepG2 cell line, derived from human hepatocarcinoma, is widely used as an *in vitro* alternative to primary human hepatocytes. HepG2 cells retain several specialised functions of normal human hepatocytes, including the expression of liver enzymes, and are considered a suitable model for *in vitro* studies of xenobiotic metabolism and liver toxicity (*44*, *45*).

The aqueous and ethanolic extracts of Caramunich III, Carafa III and Carapils showed cytotoxic effects only at a concentration of 500 µg/mL, with the exception of the aqueous extract of Carafa III after 24 h of incubation, which showed cytotoxicity from 250 µg/mL (Fig. 1) (*42*, *43*). On the other hand, the aqueous and ethanolic extracts of Pilsner showed no significant cytotoxicity at any of the concentrations tested, regardless of incubation time (Fig. 2a and Fig. 2b).

When analysing the effect of malt extracts on cell viability, it was found that the aqueous and ethanolic extracts of Carafa III (24 and 48 h) and Caramunich III (48 h) did not promote any significant increase in cell viability compared to the control at any of the concentrations tested. In contrast, in the remaining samples at least one concentration produced a significant increase (p*<*0.05) in cell viability compared to the control, which suggests a possible protective or stimulating effect on cell metabolism exerted by certain compounds present.

In general, the ethanolic malt extracts showed higher cell viability than the corresponding aqueous extracts. However, an exception was observed for the aqueous extract of Pilsner after 48 h of incubation, which showed clear superiority over the ethanolic extract. In this case, cell viability with the aqueous extract ranged from 96.3 to 312.8 %, while with the ethanolic extract it ranged from 86.2 to 206.1 %. These results suggest that, specifically for Pilsner malt, the aqueous solvent may have favoured the extraction of compounds with greater protective potential, thus promoting a more beneficial cellular response.

These results are consistent with the work of Yao *et al.* (*46*), which showed that highland barley extracts exert lipid-lowering effects on HepG2 cells, but that concentrations above 1000 µg/mL significantly compromise cell viability, suggesting a maximum concentration limit for maintaining the integrity and survival of liver cells *in vitro*.

The cytotoxicity assessment of IS-N craft beer showed that cell viability in samples with removed alcohol (ethanol, aqueous samples) ranged from 98.3 to 114.7 % after 24 h and from 86.5 to 113.4 % after 48 h of incubation, while for samples with *φ*(ethanol)=8.5 % (ethanolic samples) and the range was from 94.0 to 121.0 %, *i.e.* consistently above 80 % under the reported conditions (Fig. 2c and Fig. 2d) (*42*, *43*).

IS-N beer with or without ethanol increased cell viability compared to the control up to a concentration of 25 mg/mL after incubation for 24 (p*<*0.01) and 48 h (p*<*0.001).

However, after 24 h of incubation, IS-N beer ethanolic sample, at a concentration of 250 µg/mL, significantly reduced cell viability compared to the control (p*<*0.01), and also showed a significant decrease compared to the same concentration in aqueous sample(p*<*0.001). At 500 µg/mL, there was an even greater reduction in cell viability (p*<*0.0001), indicating cytotoxicity, as cell viability was below 80 % (*42*, *43*).

After 48 h of incubation, IS-N beer aqueous sample at 500 µg/mL also significantly reduced cell viability compared to the control (p*<*0.0001), without cytotoxicity (Fig. 2d) (*42*, *43*). This reduction in cell viability was less pronounced than that observed for the same concentration of IS-N ethanolic sample (p*<*0.05).

The increase in cell viability observed in the presence of ethanol at certain concentrations can be explained by the hormesis phenomenon associated with moderate beer consumption. This effect reflects the ability of low volume fractions of ethanol to exert beneficial actions without inducing oxidative stress, unlike excessive exposure (*47*, *48*). Furthermore, the phenolic compounds present in beer, recognised for their antioxidant and bioactive properties, may play an additional protective role in liver cells, especially under limited exposure to ethanol (*47*, *48*).

#### Enzyme alanine aminotransferase

The enzymes alanine aminotransferase (ALT) and aspartate aminotransferase (AST) are widely used as biochemical markers of liver damage, given their high amount in the liver (*14*, *49*). ALT is found predominantly in the cytosol of hepatocytes, while AST is mainly a microsomal enzyme. Increased levels of these aminotransferases in the extracellular environment indicate a hepatocellular pattern of damage, generally associated with plasma membrane injury or hepatocyte death. However, AST is less specific to the liver, as it is also present in extrahepatic tissues such as skeletal muscle, myocardium and kidneys. For this reason, although both are useful in assessing liver cytotoxicity, ALT is considered a more sensitive and specific marker for identifying direct liver damage (*14*, *49*). Fig. 3 shows the results for ALT amounts after 24 and 48 h of incubation of HepG2 cells with the samples under study.

Analysis of the concentrations at which the highest and lowest HepG2 cell viability was observed for each sample showed a clear dose-dependent relationship between ALT amounts and the concentrations of the tested samples (Table S1). The lower concentrations induced significantly reduced ALT amounts when compared to the same sample at 500 µg/mL (p*<*0.0001). The significant increase in ALT activity at 500 µg/mL suggests a more pronounced cytotoxic effect associated with the higher concentrations.

After 48 h of incubation, a further increase in ALT amounts was observed in most samples, with the exception of the aqueous extracts of Carafa III (25 µg/mL) and Pilsner (500 µg/mL), and the ethanolic extract of Carafa III (100 µg/mL). These results may indicate a possible protective effect or slower toxicity kinetics associated with certain compounds present in these formulations.

When comparing samples tested at 500 µg/mL, the ethanolic extract of Carafa III stood out, inducing the smallest increase in ALT, both after 24 and 48 h of incubation. In contrast, the aqueous extract of Carapils caused the greatest increase in enzymatic activity at the same concentration.

In general, the ethanolic extracts induced lower ALT amounts than the corresponding aqueous extracts at 500 µg/mL, except for IS-N beer ethanolic sample and Pilsner malt after 48 h, where the ethanolic extracts induced higher amounts. These results suggest that the solvent can influence the extraction of compounds with different toxicity potentials.

Although the aqueous and ethanolic extracts of Pilsner at 500 µg/mL promoted greater cell viability, this result did not translate into lower ALT activity. On the contrary, ALT amounts increased compared to the lower concentrations, at which, paradoxically, cell viability was lower.

Finally, IS-N craft beer induced lower ALT amounts than those observed in single malt extracts such as Carapils and Pilsner. This effect may be related to the presence of other typical beer components, such as hops and fermentation products, which together can modulate and attenuate the toxic effects of individual ingredients.

The literature has demonstrated the hepatoprotective potential of extracts derived from barley, although largely through experimental models other than the one used in this study. Hosseini *et al.* (*49*) evaluated the effect of aqueous barley extract on rats fed a high-fat diet. The results revealed a significant reduction in the amount of the AST and ALT enzymes (p*<*0.05) in the experimental group, indicating a beneficial effect of barley extracts in reducing the risk of fatty liver disease.

Quan *et al.* (*30*) demonstrated that pretreatment with free phenolic extract of barley (FPEB) exerted a significant protective effect in *in vivo* and *in vitro* models. In rats exposed to carbon tetrachloride, FPEB significantly reduced serum amounts of the enzymes ALT and AST (p*<*0.05), while promoting an increase in hepatic antioxidant enzymes such as superoxide dismutase, catalase and glutathione peroxidase. In addition, in buffalo rat liver (BRL) cells (rat hepatocytes) treated with CCl_4_, FPEB significantly attenuated ALT and AST amounts (p*<*0.001), as well as reducing apoptosis and induced cell damage. These results reinforce the hepatoprotective potential of barley phenolic compounds.

Park *et al.* (*50*) evaluated the effects of barley sprout extract supplementation in individuals with fatty liver induced by habitual alcohol consumption. After 12 weeks of supplementation, a significant reduction was observed in liver fat content (p*<*0.001) and in amounts of the liver enzyme γ-glutamyl transpeptidase (GGT) (p*<*0.05), indicators of improved liver function. Although the reductions in ALT and AST amounts did not reach statistical significance, there was a downward trend (ALT: (37.8±2.2) to (35.9±2.3) IU/L), suggesting a possible beneficial effect of barley sprout extract in modulating the response of the liver to alcohol-induced chronic oxidative stress.

Therefore, these results corroborate the relevance of barley-derived compounds in modulating the hepatocellular response. The lower release of ALT observed at the lowest concentrations tested, and in IS-N craft beer than in single malts, suggests that phenolic compounds and other bioactive compounds present in malt may confer properties with potential benefits for liver health, or at least be less toxic to human liver cells (*30*, *34*).

## CONCLUSIONS

Malt is the second most abundant raw material in beer and is the main source of fermentable sugars, phenolic compounds and other substances with antioxidant potential. This study found that total phenolic content (TPC) and antioxidant activity were influenced by both the variety of malt and the type of solvent used in the extraction process. IS-N craft beer showed lower TPC and antioxidant activity values than most of the malt extracts analysed. However, most of the dark malts had a higher TPC and greater antioxidant potential, especially in the aqueous extracts.

Regarding metabolic activity, the malt extracts showed no cytotoxicity up to a concentration of 250 µg/mL, while IS-N beer with 8.5 % ethanol presented cytotoxicity at a concentration of 500 µg/mL after 24 h of incubation.

In addition, incubation of HepG2 cells with IS-N beer resulted in lower amounts of the ALT enzyme than in single malts, despite its lower phenolic and antioxidant content. These results suggest a possible synergistic effect between the various bioactive compounds in beer, which could mitigate the toxic effects of the individual ingredients.

In summary, prior assessment of the biological activity of malts can enable careful selection of varieties for beer production, thereby optimising not only the antioxidant and functional profile of the beverage, but also mitigating potential adverse effects on liver function. This optimisation can be achieved by using malts with a higher content of phenolic compounds and greater antioxidant capacity, especially dark malts, which have shown better performance in *in vitro* assays, and by the appropriate choice of solvents and extraction conditions, favouring the incorporation of more stable and biologically relevant bioactive compounds in the final product. However, to confirm further these observations, it will be important to expand the analysis to different beer styles and apply complementary extraction and antioxidant methods, in addition to performing phytochemical characterisation of the samples and developing *in vivo* studies to assess the bioavailability, efficacy and safety of the identified compounds. These advances will allow for a more comprehensive understanding of the functional potential of craft beer and validate the real impact of its antioxidant and cytoprotective properties on liver health.

## SUPPLEMENTARY MATERIALS

Supplementary materials are available at: https://www.ftb.com.hr/images/pdfarticles/2026/July-September/FTB-64-311-S1.pdf.

## Appendix

**Table S1:** Results of determination of ALT of different concentrations of IS-N beer and aqueous and ethanolic extracts of malts, in HepG2 cells

		*t*(incubation)/h	
Malt extract	*γ*/(μg/mL)	24	48
		ALT/(IU/L)	
Carafa III aqueous	25	(2.57±0.09)	(2.00±0.66)
	500	(15.4±0.4)	(16.7±0.8)
Carafa III ethanolic	100	(2.9±0.7)	(2.8±0.8)
	500	(10.0±0.7)	(13.2±0.5)
Caramunich III aqueous	50	(2.0±0.3)	(3.93±0.4)
	500	(16.0±0.9)	(18.0±1.0)
Caramunich III ethanolic	50	(3.1±0.8)	(3.8±0.2)
	500	(15.49±0.00)	(16.6±1.4)
Carapils aqueous	100	(0.00±0.00)	(2.2±0.7)
	500	(25.9±0.8)	(27.2±0.6)
Carapils ethanolic	10	(1.3±0.2)	(3.2±0.6)
	500	(18.04±0.47)	(21.2±0.3)
Pilsner aqueous	1	(0.00±0.00)	(5.1±0.2)
	500	(10.21±0.00)	(18.8±1.6)
Pilsner ethanolic	1	(1.5±0.2)	(4.1±0.7)
	500	(17.9± 0.2)	(19.3±1.1)
IS-N (aqueous sample)	1	(0.68±0.09)	(1.43±0.09)
	500	(12.7±1.1)	(14.4±0.8)
IS-N (ethanolic sample)	1	(1.5±0.2)	(4.0±0.4)
	500	(17.4±0.8)	(18.7±0.6)

IS-N (aqueous sample)=beer with removed ethanol, IS-N (ethanolic sample)=beer with *φ*(ethanol)=8.5 %
